# An assessment of mutagenicity of chemical substances by (quantitative) structure–activity relationship

**DOI:** 10.1186/s41021-020-00163-1

**Published:** 2020-07-02

**Authors:** Masamitsu Honma

**Affiliations:** National Institute of Health Sciences, 3-25-26 Tonomachi, Kawasaki-Ku, Kanagawa 210-9501 Japan

**Keywords:** (quantitative) structure–activity relationship ((Q)SAR), Ames test, ICH-M7, Rule-based, Statistics-based, Prediction power, Benchmark data set, Deep leaning, AI

## Abstract

Currently, there are more than 100,000 industrial chemicals substances produced and present in our living environments. Some of them may have adverse effects on human health. Given the rapid expansion in the number of industrial chemicals, international organizations and regulatory authorities have expressed the need for effective screening tools to promptly and accurately identify chemical substances with potential adverse effects without conducting actual toxicological studies. (Quantitative) Structure–Activity Relationship ((Q)SAR) is a promising approach to predict the potential adverse effects of a chemical on the basis of its chemical structure. Significant effort has been devoted to the development of (Q) SAR models for predicting Ames mutagenicity, among other toxicological endpoints, owing to the significant amount of the necessary Ames test data that have already been accumulated. The International Council for Harmonisation of Technical Requirements for Pharmaceuticals for Human Use (ICH) M7 guideline for the assessment and control of mutagenic impurities in pharmaceuticals was established in 2014. It is the first international guideline that addresses the use of (Q) SAR instead of actual toxicological studies for human health assessment. Therefore, (Q) SAR for Ames mutagenicity now require higher predictive power for identifying mutagenic chemicals. This review introduces the advantages and features of (Q)SAR. Several (Q) SAR tools for predicting Ames mutagenicity and approaches to improve (Q) SAR models are also reviewed. Finally, I mention the future of (Q) SAR and other advanced in silico technology in genetic toxicology.

## Introduction

In July 2015, the Chemical Abstracts Service (CAS), a global authority on chemical information, and the information division of the American Chemical Society announced the registration of the one hundred-millionth chemical substance to its chemical substance database. Only five years later, the number of registered chemical substances is about to surpass 200 million (https://www.cas.org/). This means that a new chemical substance was registered every two minutes, and as this pace would accelerate in the future, the number is expected to surpass one billion 10 years from now. Currently, of these chemical substances, approximately 100,000 types of chemical substances are industrially produced and exist in our living environment, and this number is also growing (https://www.minambiente.it/sites/default/files/archivio/allegati/reach/InterpretationWSSDGoals.pdf). These chemical substances released into the environment, some may adversely affect human health or affect the global environment or ecosystem, for instance, by causing global warning or destroying natural resources. Therefore, there is a need to appropriately assess and manage these chemical substances. In World Summit on Sustainable Development (WSSD) held in 2002 in Johannesburg, South Africa, there was an agreement on (and later expanded), “aiming to achieve, by 2020, that chemicals are used and produced in ways that lead to the minimization of significant adverse effects on human health and the environment, using transparent science-based risk assessment procedures and science-based risk management procedures, taking into account the precautionary approach” (WSSD 2020 Goal). Regulatory authorities in developed countries are being requested to endeavor to achieve the WSSD Goal. In Japan, based on the “Act on the Regulation of Manufacture and Evaluation of Chemical Substances (Chemical Substances Control Law)” established in 1968, the characteristics (degradability and accumulative properties) and effect of new chemical substances on the environment and human health are examined, and necessary regulation regarding their production, import, use, etc., has already been implemented. Based on the Chemical Substances Control Law, tens of thousands of new chemical substances are registered annually. However, at present, there are only approximately 400 mass-produced chemical substances, whose production/import volume exceeds 10 tons, and they are investigated by the joint council, which comprises the Japanese Ministry of Economy, Trade and Industry, the Ministry of the Environment, and the Ministry of Health, Labor, and Welfare (https://www.meti.go.jp/policy/chemical_management/english/cscl/). A large number of other chemical substances are registered without considering their effect on the environment or humans health assessed.

The safety of chemical substances is typically assessed by biological tests using animals, mammalian cells, microorganisms, etc. However, testing such a vast quantity of chemical substances in this manner is unrealistic when considering labor, time, cost, and animal welfare. To achieve the WSSD Goal, an effective screening tool capable of promptly and accurately identifying harmful chemical substances is required. (Quantitative) Structure–Activity Relationship ((Q)SAR) is an in silico method for predicting chemical substances causing adverse effects on the basis of their chemical structures. Currently, under the Chemical Substance Control Law, each ministry is employing (Q) SAR as a tool to predict adverse effects. The related prediction results, however, are provided to the committee only as a reference, and have not yet reached to the practical application instead of actual experimental studies (Table 1).

**Table 1:** (Q) SAR tools used in Chemical Substance Control Law in Japan

	Evaluation item	QSAR tools
Ministry of Economy, Trade and Industry	Degradability	BIOWIN5
		BIOWIN6
		CATALOGIC
	Accumulation	BCFWIN
		Amot-Gobas Model
		Baseline Model
Ministry of the Environment	Ecological effects	TIMES
		ECOSAR
		KATE
Ministry of Health, Labor and Welfare	Human health effects (Ames mutagenicity)	DerekNexus
		CASE Ultra
		TIMES_Ames

## Mutagenicity and (Q)SAR

Regarding the toxicity of chemical substances, many substances have a level below which toxic effects are not observed, i.e., a threshold. Thus, if the exposure level is sufficiently lower than the threshold, health risks from the substance’s toxicity can be considered to be zero. It is Acceptable Daily Intake (ADI) or Permitted Daily Exposure (PDE). On the other hand, among the many types of toxicity, mutagenicity is a key mechanism in oncogenic processes via chemical substances; it is based on the chemical reactivity between DNA and chemical substances resulting in mutations. Mutation is an irreversible and permanent change. Even one mutation in genome has the possibility of generating cancerous cell; therefore, a threshold value cannot be assigned. There is another term called “genotoxicity,” which has a broader definition than mutagenicity. A genotoxic substance damages the DNA or chromosome, causing structural or quantity-related genome changes, but does not always induce a mutation. A substance that is genotoxic but not mutagenic has the ability to damage DNA, but there is no direct evidence that it will cause permanent genetic change related to carcinogenesis. It has also been stated that threshold values can be set for genotoxic substances that are not mutagenic [1]. Therefore, even at low exposure levels, the assessment of mutagenicity, its presence or absence in particular, is important for assessing the cancer risk of chemical substances. The Ames test, one of the typical mutagenicity tests, is consistently required for testing the safety of chemical substances where exposure is extremely low (e.g., residual agricultural chemicals or food additives that are found in trace amounts in food products, eluates from plastic containers, or impurities found in pharmaceuticals).

In this review, I am mainly focusing on (Q) SAR for the Ames test. In the field of (Q) SAR related to the assessment of chemical substances’ effects on human health, studies on the Ames mutagenicity prediction (Q) SAR are the most advanced, and the method has already been in practical use. The reasons for such advancement, in addition to the aforementioned importance of the Ames test, are:

1. Mutagenic chemical substances generally have electrophilic chemical structures, which provide molecular mechanisms whereby mutagenicity can be explained using physical chemistry [2].
2. Results from the Ames test have well reproducibility (80–85%) and are consistent in comparison with results from other toxicological tests [2]. Of all the toxicity tests, the Ames test has the greatest quantity of accumulated tested data. This large set of reliable test data makes it easy to develop a (Q) SAR prediction model.
3. The International Council for Harmonisation of Technical Requirements for Pharmaceuticals for Human Use guideline for the assessment and control of mutagenic impurities in pharmaceuticals (ICH-M7) was established in 2014. This guideline recommends the use of (Q) SAR for initially assessing the mutagenicity of impurities in pharmaceuticals instead of actual Ames tests [3]. This guideline has greatly promoted the development of (Q) SAR models for predicting Ames mutagenicity in the last several years.

(Q) SAR is the study of the correlation between chemical structure and toxicity. Toxicity of a chemical substance generally has a quantitative manner, so the original purpose of toxicity prediction using (Q) SAR is to predict the dose at which an effect would be observed (the end-point). Meanwhile, in mutagenicity tests (genotoxicity tests) such as the Ames test, the assessment is not done quantitatively, but rather, the results are binary, pertaining to the presence or absence (positive or negative) of mutagenicity. That such qualitative results facilitate easy validation (either correct or incorrect) of the prediction model. It is another reason of the advancement of easily modeling the Ames mutagenicity prediction (Q)SAR. Thus, the Ames mutagenicity prediction is originally SAR, which is why I attach the letter Q in parentheses. In this review, for convenience, I will hereinafter use the expression QSAR.

## Prediction of Ames mutagenicity by QSAR

**Fig. 1:**
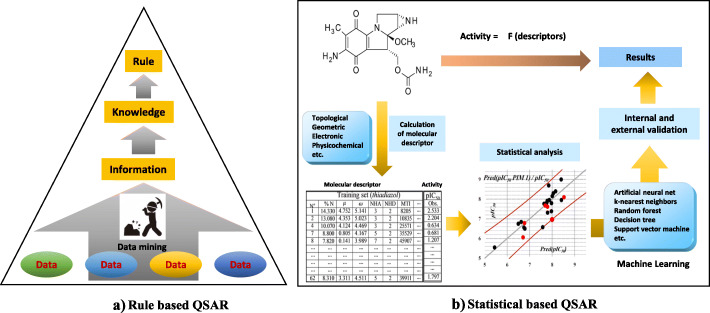
Rule based QSAR and statistical based QSAR

Studies for predicting mutagenicity or carcinogenicity from the structure of chemical substances have been being conducted since a long time. In the 1960s, James & Elisabeth Miller et al. [4] focused on the electrophilicity of carcinogenic alkylators. They proposed an electrophilic theory, stating that many carcinogenic chemical substances are electrophilic derivatives, or that they are metabolized to become such derivatives in human body, and bond with nucleophilic groups such as DNA or proteins in the target tissue for carcinogenesis, causing cancer. Since then, the study of chemical carcinogenesis has progressed rapidly. Bruce Ames developed a series of salmonella strains sensitive to carcinogenic chemical substances, e.g., alkylators and intercalators, and established the Ames test [5–7]. The Ames test can be regarded as an in vitro model for detecting carcinogenic chemical substances. Most of the carcinogenic substances from the mutational mechanism gave positive results in the Ames test, and these results were considered valid in the scope of Millers’ hypothesis. Following Miller’s electrophilic theory, John Ashby and Raymond Tennant developed a structural alert (SA) and carcinogenicity prediction compilation for carcinogenic chemical substances [8, 9]. Carcinogenic SA was defined as a molecular functional group or substructure related to the carcinogenic activity of chemical substances. It was also considered as a mutagenic SA that induces gene mutation, which is an important step in carcinogenesis. Ashby identified, out of 222 chemical substances in the U.S. National Toxicology Program, 18 types of SAs that showed a strong correlation with positive results in rodent carcinogenicity testing [10]. Currently, there are two main types of Ames mutagenicity QSAR models: rule-based and statistics-based (Fig. 1). The rule-based QSAR, as first implemented by Ashby et al., is a method where characteristic substructures that give positive results are defined based on already known data, and Ames tests results are predicted qualitatively using an established empirical rule. On the other hand, statistics-based QSAR is based on physicochemical properties expressed in terms of molecular descriptors (numerical data) such as geometric, electronic, physicochemical, and descriptors that correlate highly with positive results in the Ames test and are used to predict test results using machine learning methods.

In the past, regarding Ames mutagenicity QSAR, many QSAR tools have been developed for both academic and commercial purposes. The Organisation for Economic Cooperation and Development (OECD) indicated that QSAR will be used actively in future toxicity assessments of chemical substances and published principles for international harmonization in relation to QSAR assessment (Table 2). Currently, these principles are not mandatory and have been delegated to their respective countries.

**Table 2:** OECD Principles for the validation, for regulatory purposes, of QSAR models

**1. A defined endpoint**	
Clarify the endpoint of a test system for the predictive model (predict Ames test results, chromosomal aberration test results, not predict genotoxicity or mutagenicity).	
**2. An unambiguous algorithm**	
Clarify the types of models (rule-based and statistical-based) and the methods (algorithms, descriptors, etc.) used to build the models, and ensure their transparency. However, in the case of models for commercial purposes, this information is often not necessarily disclosed.	
**3. A defined domain of applicability**	
Since the predictability of QSAR depends on the training set used to build the model, the types of chemicals that can make highly accurate predictions are limited. Therefore, clarify the limits of the chemical structure to which the QSAR model can be applied (Clarification of Out of Domain).	
**4. Appropriate measures of goodness-of–fit, robustness and predictivity**	
The fitness and robustness of the predictive model should be evaluated using an internal training set. Also, its predictability should be determined using an appropriate external dataset.	
**5. A mechanistic interpretation, if possible**	
If possible, show the mechanical association between the model descriptor and the prediction endpoint. If it can be interpreted by mechanisms, it can be part of the scope of Principle-3.	

## Leading QSAR tools for predicting Ames mutagenicity

### Derek Nexus (Lhasa limited, UK): rule-based QSAR

Derek (Deductive estimation of risk from existing knowledge) Nexus is a rule-based expert QSAR system available commercially as part of the Lhasa Knowledge Suite. The knowledge rules included are created with consideration of insights related to SA, chemical compound examples, and metabolic activation and mechanism. These knowledge rules are being continuously developed by the provision of data and knowledge by private corporations, universities, public research institutions, and non-profit organizations. In Derek Nexus, a prediction is derived by comparing the structural characteristics of target compounds that have toxicophores (in other words, SAs that are assumed to be involved in toxic actions) that are coded as structural patterns in its knowledge base. The final prediction is derived from the existence of toxicophores in the queried structure. The validity of the Derek Nexus prediction is usually confirmed by referencing related literature, and the user can obtain a reliable prediction. The main strength of Derek Nexus is transparency of prediction, the fact that user groups give their assessments during rule development, and the ease with which new rules can be added. As this system is a rule-based system, there is no prescribed training set or scope of applications. However, using the recently included structure classification function, it has become possible for users to verify their negative result predictions [11]. In this system, chemical compounds that have alerts with an inference level of equivocal or higher are processed as positive result predictions.

### Sarah Nexus (Lhasa limited, UK): statistics-based QSAR

The Lhasa Knowledge Suite also includes a statistics-based Ames mutagenicity model called the Sarah Nexus. In this model, a self-organizing hypothetical network (SOHN) is used to produce an activity hypothesis for queried compounds based on the existence of substructures, whose relation to activation or deactivation in the training set has been acknowledged [12]. Hypotheses are integrated, and a comprehensive prediction is made regarding the activity of queried compounds. In addition, the reliability of each prediction is shown. Predictions are determined by the similarity of queried compounds and the nearest data in the training set for each hypothesis. Next, the reliability of each individual hypothesis is integrated, and the reliability of the comprehensive prediction and prediction for the queried compound is obtained (This comprehensive prediction may be positive, negative, equivocal, or outside the applicable scope). For the user, in addition to a comprehensive prediction, hypotheses and examples from related training sets are displayed, along with related metadata.

### CASE ultra (MultiCASE Inc., USA): statistics-based QSAR

CASE Ultra is a toxicity prediction software based on QSAR developed by MultiCASE Inc. in the United States. CASE Ultra uses a statistical method as its basis, and alerts are automatically extracted from training data using machine learning technology. The data required for training are the chemical structures and their toxicity labels [13, 14]. The degree of toxicity predicted for the queried chemical substance depends not only on the specified alert, but also on the structural environment around the alert. The structural characteristic of the alert surroundings is called the “modulator,” and this is also learned automatically from the training data. In this algorithm, to construct a QSAR model with continuous toxicity endpoints, various physical chemistry parameters and descriptors are used. CASE Ultra’s main models that relate to Ames mutagenicity comprise four modules: GT1_AT_ECOLI, GT1_A7B, PHARM_ECOLI, and PHARM_SALM. In 2018, GT1_BMUT, the consensus model for *Salmonella/E. coli* was released.

### Leadscope model applier; LSMA (Leadscope Inc., USA): statistics-based QSAR

This QSAR model for mutagenicity is a statistics-based QSAR tool developed by Leadscope Inc. in collaboration with the U.S. Food and Drug Administration. The models employed are constructed from training sets of publicly released Ames mutagenicity data, and the structural descriptors are based on the following: (1) pre-defined structural characteristics, (2) automatically generated chemical frameworks, (3) external knowledge, and (4) computed characteristics. Selected descriptors (independent variables) and Ames mutagenicity data (responding variables) are used to predict Ames test results using a partial logistic regression model. The model displays the probability of obtaining a positive result and structural characteristics contributing to activation and deactivation. It also displays detailed test information related to the chemical substances in the training set. This enables experts to conduct detailed reviews of the training set.

### TIMES_AMES (Bourgas University, Bulgaria): rule-based QSAR

The TIMES_AMES QSAR tool is included in the OASIS/TIMES software, which is commercially available and provided by Bourgas University. The Ames/QSAR tool includes SA, modifiers for explaining other effects related to molecular structure, and an interaction mechanism between DNA and SA. A rule base is used to determine SA, and pattern recognition (Mechanism-based common reactivity pattern) is used for mutagenicity prediction. OASIS/TIMES is equipped with a liver metabolism simulator based on metabolic pathways (Tissue Metabolite Simulator; TIMES). Chemical substances in the training set used in this model can be classified into those that have mutagenicity without metabolic activation, those that have mutagenicity after metabolic activation, and those that are not mutagenic regardless of metabolic activation [15, 16]. When the Ames mutagenicity under the presence of rat S9 is predicted for queried compounds, the metabolite predicted to have mutagenicity is displayed, along with a metabolic map of the compound. This is OASIS/TIMES’ greatest advantage.

### Toxtree (Istituto Superiore di Sanità, Italy): rule-based QSAR

ToxTree is a Java-based open source application that categorizes chemical substances and predicts their toxicity using a decision tree approach. ToxTree was developed by Ideaconsult Ltd. (Bulgaria) pursuant to a contract with the European Commission’s Joint Research Center. ToxTree, freely available to all, is a service designed for scientific researchers and other individuals. The Benigni/Bossa rule is used for predicting Ames mutagenicity [17, 18]. Similar to Derek, it is theoretically difficult to assess a chemical substance that does not have a negative SA.

### ADMEWORKS (FUJITSU KYUSHU SYSTEMS LIMITED, Japan): statistics-based QSAR

ADMEWORKS is a consensus model comprising two statistical models (one with high sensitivity and one with high specificity) developed by Fujitsu Kyushu Systems Ltd. It was mainly developed using 1977 types of chemical compounds obtained from the U.S. National Toxicology Program (NTP). In the case where results from the two statistical models coincide, i.e., both being positive or negative, the final estimation result is displayed. Otherwise a prediction will not be made. In addition to the consensus model, positive SAs and negative SAs are extracted from the original data and used as filters before a prediction is made using the consensus model. If the chemical compound includes any positive alert, it is immediately classified as *positive*; if it includes any negative alert, it is immediately classified as *negative*. If no alert is included in the compound, the consensus model will be used to perform a final prediction.

### ChemTunes•ToxGPS (molecular networks GmbH and Altamira LLC, USA): statistics-based QSAR

The ChemTunes model is based on a ToxGPS knowledge base of in vivo and in vitro toxicity data collected from regulation-related literature and primary information sources. Predictions are performed using a QSAR model that is based on action mechanisms and “chemical species” alerts that are integrated using a “weight of evidence” method for quantitative evidence, based on the Dempser–Shafer theory. In this model, a structure base (ToxPrint chemical species, etc.), a characteristics base (dipole moments, solubility, logP, etc.), and quantum descriptors are used. The ChemTunes•ToxGPS model can be used for various human health-related toxicity endpoints. The deterministic approach estimates uncertainty, and it is described by combining predictions from multiple models and takes into consideration the reliability of the information source for each piece of evidence. Although not described here, the ChemTunes•ToxGPS prediction system also prepares estimate values for the uncertainty in each prediction.

### MUT_Risk (simulations plus Inc., USA): statistics-based QSAR

MUT Risk is an ADMET Risk™ score that summarizes the mutagenicity predictions using 10 separate Artificial Neural Networks Ensemble classification models (results of 10 individual Ames tests with 5 strains under the presence or absence of rat S9). For each positive classification by each of five ±S9 model pairs, a point is added to a total score. A threshold value used for judgment is set by a user. MUT_Risk-0 judges whether the chemical compounds are mutagenic when the score is greater than 0, while MUT_Risk-1 judges whether the compounds are mutagenic when the score is greater than 1. This approach allows the tradeoff between sensitivity and specificity to be adjusted in response to each application. Each of the 10 contributing models has its own unique out-of-scope flags and estimated uncertainty values.

### StarDrop auto-Modeller (Optibrium ltd., UK) statistics-based

StarDrop is an integrated QSAR tool; it features Derek Nexus as a knowledge-based toxicity prediction function and Auto-Modeller as a statistics-based prediction function. In Auto-Modeller, a data set that includes structural information and the physical property values of prediction targets allows the creation of an original prediction formula almost automatically. This means that there is no need to prepare descriptor information, and the Modeller includes a function to automatically generate nine types of descriptors related to whole molecules (molecular weight, logP prediction values, etc.) and 321 types of descriptors in SMARTS format (the number of atomic species, characteristic group, etc.). It is also easy to add original descriptors, and the application range is wide. In this analytical method, 10 types of continuous models and three types of category models are included, and a function for automatically judging the best model is also included. The high level of automation of such high-quality capability of processing structural information and statistical processing supports not only non-specialists in the development of physical property prediction models, but also specialists in streamlining their trial-and-error processes.

### T.E.S.T. (US Environmental Protection Agency, USA): consensus model

The Toxicity Estimation Software Tool (T.E.S.T) is free software from the US Environmental Protection Agency that can predict endpoints for three human health effects, including Ames mutagenicity. The T.E.S.T. was built with the belief that providing predicted results from different algorithms would make the predicted toxicity more reliable. Therefore, T.E.S.T. includes a plurality of prediction models constructed by a plurality of algorithms using the same data set [19]. Regarding the prediction of Ames mutagenicity, models using the following four types of algorithms are installed in T.E.S.T.

1. Hierarchical method: Statistics-based QSAR using hierarchical clustering method
2. FDA method: Statistics-based QSAR using multiple regression analysis for collected similar substances
3. Nearest neighbor method: Statistics-based QSAR using the average data of three similar substances collected from the training data set
4. Consensus method: The values obtained by the above method are averaged.

### VEGA (Istituto di Ricerche Farmacologiche Mario Negri, Italy): consensus model

VEGA is a Java-based system developed by Italy’s Istituto di Ricerche Farmacologiche Mario Negri and is available for free download. VEGA includes multiple QSAR models developed using different data sets, and its calculation results are outputted by the same display method. The *Mutagenicity (Ames test) model* from the most recent VEGA version 1.1.4 includes the following four models:

1. CAESAR: Statistics-based QSAR using a support vector machine (SVM)
2. SarPy: Rule-based QSAR based on substructures
3. ISS: Rule-based QSAR based on Benigni/Bossa rules
4. KNN: Analogy (read-across) using istKNN

Ultimately, a consensus model derives a result based on the results from these four models.

## Assessing the predictive power of QSAR tools

Because the Ames test result are binary—positive or negative—their predictive power can be objectively quantified and assessed from their coincidence with calculation results from QSAR. The 2 × 2 prediction matrix comprising true positive (TP), false positive (FP), false negative (FN), and true negative (TN) is given in Table 3. Calculation methods for sensitivity (ability to detect positive substances), specificity (ability to detect negative substances), prediction rate of positive and negative (accuracy of predictions), accuracy, balance accuracy, applicable scope, and Mathews’ correlation coefficient (MCC) are given in Table 4. For high-quality QSAR models, high sensitivity (surely detecting mutagenic substances), high negative prediction value (the one predicted as negative is indeed negative), high coverage (able to evaluate as many chemical substances as possible) are considered important. These numerical values depend highly on the balance of the data set being assessed, and thus caution is required. Data sets subjected to the assessment typically have a significant amount of negative data. In the case where the ratios of positive and negative in the data set are 10 and 90%, respectively, even a QSAR model with a sensitivity of 40% can be considered a good model. Balance accuracy and MCC are predictive indicators, taking into consideration a data set’s bias, and are useful for assessing the QSAR tool’s predictive power using data sets from different assessment subjects.

**Table 3:** 2X2 prediction matrix for Ames mutagenicity classification

		Experimental Ames mutagenicity class	
		Positive	Negative
QSAR prediction class	Positive	True Positive (TP)	False Positive (FP)
	Negative	False Negative (FN)	True Negative (TN)
	Unpredictable (OOD^a^)	–	–

^a^Out of Domain

**Table 4:** Performance metrics used to evaluate classifiers

Performance metric	Calculation and description
Sensitivity (SENS)	*TP*/(*TP* + *FN*)
	Measures the ability of a QSAR tool to detect Ames positives compounds correctly.
Specificity (SPEC)	*TN*/(*TN* + *FN*)
	Measures the ability for a QSAR tool to detect negatives compounds.
Accuracy (ACC)	(*TP* + *TN*)/(*TP* + *TN* + *FP* + *FN*)
	Assesses a QSAR tool’s overall performance by returning the fraction of compounds which were correctly predicted.
Balanced Accuracy (BA)	(*SENS* + *SPEC*)/2
	Assesses the overall model performance, giving each class equal weight.
Positive Prediction Value (PPV)	(*TP*)/(*TP* + *FP*)
	Indicates how frequently positive predictions are correct.
Negative Prediction Value (NPV)	*TN*/(*TN* + *FN*)
	Indicates how often negative predictions are correct.
Mathews Correlation Coefficient (MCC)	$\frac{\left(\left( TP\ast TN\right)-\left( FP\ast FN\right)\right)}{\sqrt{\left( TP+ FP\left)\left( TP+ FN\right)\left( TN+ FP\right)\right( TN+ FN\right)}}$
	Assesses the overall performance of the model. Values can range from −1 to 1, which is in contrast to the other metrics in this table which range form 0 to 1.
Coverage (COV)	(*TP* + *TN* + *FP* + *FN*)/(*TP* + *TN* + *FP* + *FN* + *OOD*)
	Evaluates the proportion of compounds for which the model can make a positive or negative prediction.

## The application of QSAR in ICH-M7

ICH-M7 guideline for the assessment and control of mutagenic impurities in pharmaceuticals was established in 2014. This was the first international guideline that allowed QSAR to be used as an alternative to biological experiments to assess human health effect^3^. Impurities in pharmaceuticals are found in trace amounts, consist of many types, and in some cases they are unstable. It is therefore often impossible to assess their toxicity via isolation, purification, and biological experiments. If the chemical structure of the impurity is known, this guideline that allows the assessment of mutagenicity using QSAR is very realistic. In ICH-M7, the use of both rule-based and statistics-based QSAR tools is required for the assessment of Ames mutagenicity. Regarding these QSAR tools, as long as the aforementioned OECD validation principles are adhered to, any QSAR tool can be used. The absence of structural alerts from two QSAR methodologies (rule-based and statistical-based) is sufficient to conclude that the impurity is of no mutagenic concern, and no further testing is recommended. In cases where prediction results differ from one tool to another, or prediction could not be carried out because of out of domain, it is stated that a conclusion can be drawn based on an appropriate review by experts. However, in the guideline, nothing is specifically described regarding the specific strategy for such expert judgments. In Japan, with the Japanese Environmental Mutagen Society (JEMS), a workshop for developing these strategies of expert judgments is conducted every year [20, 21].

## Ames/QSAR international challenge project for the evaluation and improvement of QSAR tools’ predictive power

The establishment of ICH-M7 in 2014 has changed the role of QSAR from a tool for pre-screening and predicting Ames mutagenicity to a test for providing data on assessment of mutagenicity of chemical substances. Thus, greater predictive power of QSAR tools for Ames mutagenicity predictions is required. However, in practice, the prediction accuracy of many QSAR tools is insufficient. In particular, the sensitivity for new chemical substances is reported to be 50% or lower [22]. To improve the prediction accuracy of QSAR, the accumulation of actual Ames test data as training data is important. Training data that include a large amount of data on chemical substances with unique structures would expand the chemical space, reduce false positive (FP) and false negative (FN) results, and improve prediction accuracy. The reliability of such test data is also important. Currently, there are approximately 10,000 chemical substances whose Ames test results are publicly available through online websites. Many QSAR developers are developing QSAR models based on these databases [18].

In Japan, the safety of new chemical substances is assessed by industries under the Ministry of Health Labor and Welfare (MHLW) according to the Chemical Substances Control Law as well as the Industrial Safety and Health Act (ANEI-HOU). The purpose of the ANEI-HOU is to secure safety and health in the workplace. For chemical substances to be newly manufactured or imported in excess of 100 kg per year, ANEI-HOU stipulates that producers conduct hazard investigations in advance and to notify the MHLW of the results. As part of the hazard investigation, the Ames test or its equivalent (e.g. rodent carcinogenicity testing) is required. The Chemical Hazards Control Division (CHCD), Industrial Safety and Health Department, Labor Standards Bureau, MHLW of Japan is responsible for monitoring industries under the ANEI-HOU. Since ANEI-HOU started form 1979, more than 20,000 chemical substances’ Ames tests have been conducted by chemical companies, pharmaceutical companies and contract laboratories under GLP compliance. It is now the world’s largest Ames test big-data. The results of the Ames tests are confidential and cannot be basically disclosed.

The Division of Genetics and Mutagenesis, National Institute of Health Sciences of Japan (DGM/NIHS) recently established a new Ames database consisting of 12,140 new ANEI-HOU chemical substances for developing QSAR models, and organized an international AMES/QSAR Challenge Project for the evaluation and improvement of Ames/QSAR tools with the permission from the CFCD office of MHLW. The Challenge Project was conducted from 2014 to 2017 in collaboration with 12 QSAR vendors with 17 QSAR tools from the USA, UK, Italy, Spain, Bulgaria, Sweden, and Japan (Table 5). Based on the hypothesis that expansion of training data enhances the predictive power of QSAR tools, a three-phase challenge was designed. In each phase, a list of about 4000 chemicals without their Ames test results were provided to the QSAR vendors. The QSAR vendors predicted the Ames mutagenicity using their QSAR tools and reported the results to the DGM/NIHS. The DGM/NIHS validated the performance of the QSAR tools and disclosed the Ames results. Table 6 summarizes the number of Ames-positive and –negative chemicals in each phase. Interestingly, the portions of Ames-positive chemicals was constant in each phase (approximately 15%) despite the chemicals were arbitrarily divided into phases regardless of the Ames results. It means that roughly 15% of chemical substances newly appearing in commerce are Ames mutagens. This information is valuable for quality control of Ames mutagenesis datasets and for appropriate allocation of resources to assure the safety of commercial chemicals.

**Table 5:** Participants in Ames/QSAR international challenge project

QSAR Vender	QSAR Tool
1. Lhasa Limited (UK)	① Derek Nexus
	② Sarah Nexus
2. MultiCASE Inc. (USA)	③ CASE Ultra statistical-based
	④ CASE Ultra rule-based
3. Leadscope Inc. (USA)	⑤ Leadscope statistical-based
	⑥ Leadscope rule-based
4. IRCCS - Istituto di Ricerche Farmacologiche Mario Negiri (Italy)	⑦ CAESAR
	⑧ SARPY
	⑨ KNN
5. LMC - Bourgas University (Bulgaria)	⑩ TIMES_AMES
6. Istituto Superiore di Sanita (Italy)	⑪ Toxtree
7. Prous Institute (Spain)	⑫ Symmetry
8. Swedish Toxicology Science Research Center (Sweden)	⑬ AZAMES
9. FUJITSU KYUSHU SYSTEMS LIMITED (Japan)	⑭ ADMEWORKS
10. IdeaConsult Ltd. (Bulgaria)	⑮ AMBIT
11. Molecular Networks GmbH and Altamira LLC (USA)	⑯ ChemTune•ToxGPS
12. Simulations Plus, Inc. (USA)	⑰ MUT_Risk

**Table 6:** Number of chemicals in Ames/QSAR international challenge project

Class	Phase I (2014–2015)	Phase II (2015–2016)	Phase III (2016–2017)	Total (2014–2017)
**Positive**	556 (14.5%)	562 (14.7%)	629 (14.3%)	1757 (14.4%)
**Negative**	3336 (85.5%)	3267 (85.3%)	3780 (85.7%)	10,383 (85.6%)
**Total**	3902	3829	4409	12,140

As the result of three trials of the Ames/QSAR International Challenge Project, all QSAR tools were considerably improved. Most tools achieved > 50% sensitivity and accuracy was as high as 80%, which is almost equivalent to the inter-laboratory reproducibility of Ames tests (Table 7), implying that the project was successfully completed [23]. The DGM/NIHS will start the next Ames/QSAR International Challenge Project near future, because more than 2000 new chemicals’ Ames tests results submitted to ANEI-HOU has been accumulated during recent a few years.

**Table 7:** Averages and ranges of the performance metrics of QSAR tools in the Ames/QSAR challenge project

Performance metric	Phase I	Phase II	Phase III
Sensitivity (%)	56.7 (38.6–70.0)	58.0 (41.6–72.1)	57.1 (31.7–67.6)
Specificity (%)	77.7 (62.5–91.5)	84.2 (64.9–92.8)	79.9 (60.7–93.0)
Accuracy (%)	74.7 (63.6–83.9)	80.3 (65.8–87.7)	76.7 (68.0–87.3)
Balanced Accuracy (%)	67.2 (62.1–72.5)	71.1 (64.0–78.9)	68.5 (62.0–74.4)
Positive Prediction Value (%)	31.2 (24.8–43.1)	41.2 (27.4–56.3)	34.8 (21.1–51.0)
Negative Prediction Value (%)	91.5 (89.4–92.5)	91.9 (88.1–94.2)	92.0 (89.1–93.6)
MCC	0.28 (0.20–0.39)	0.37 (0.25–0.50)	0.31 (0.17–0.44)
Coverage (%)	91.4 (57.7–100)	89.1 (22.7–100)	92.3 (74.5–100)

## Construction of big-data database of chemical substances and development of new platform for human safety assessment, using AI and deep learning, for pharmaceuticals, foods, and household chemical substances

At the NIHS, we are studying toward the construction of a big-data database of chemical substances that integrates, upgrades, and expands large-scale and reliable toxicity testing data pertaining to human health that we have been consolidating over many years. From 2018, we have started research on the development of a safety prediction platform based on integrate expertise in safety evaluation, experience in high-precision safety research, and artificial intelligence (AI) such as deep learning. This platform is targeted at pharmaceuticals, foods, and other chemicals in our living environment. By integrating insights from fields with differing regulations, this is expected to contribute to the acceleration/streamlining/sophistication of toxicity assessment, creating preventative measures against side effects from pharmaceuticals, establish reliable safety-assessment standards for household and food related chemical substances, and strengthen Japan’s industrial competitiveness.

**Fig. 2:**
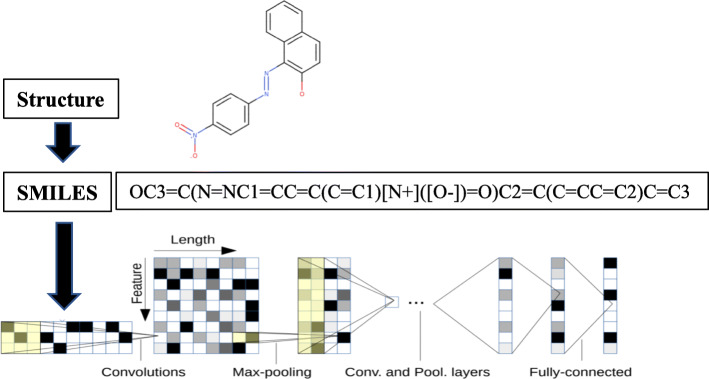
Convolutional Neural Network (CNN) from SMILES text

Of many types of toxicity testing data, because the Ames test data have the greatest quantity, we are developing an Ames test prediction model that employs deep learning in this project. In our prototypical model, feature quantities are automatically extracted from chemical structures described in the simplified molecular-input line-entry system (SMILES) notation using a convolutional neural network (CNN). Usually, a CNN is used for image classification; however, it can also be applied to text classification based on character information such as SMILES. First, a character in SMILES notation is converted into a one-hot vector with 70 dimensions. After this, normalization is performed according to the maximum number of characters; this is taken to be a two-dimensional array of “70 × maximum number of characters,” and the convolution is performed similar to the case of images. By using this method, it is possible to include information of what characters are placed before and after a character, enabling the detection of feature quantities related to chemical structure (Fig. 2). By having a SMILES-CNN model learn Ames test data for 16,651 chemical substances and then testing its accuracy using an assessment data set of 2000 new substances, we were able to obtain results with 55% sensitivity, 80% specificity, and 77% accuracy. This prediction rate is comparable with prediction rates of other QSAR tools. Currently, we are aiming to improve the prediction accuracy by enhancing the learning data set and adding explanatory variables. Apart from the use of SMILES-CNN, we are implementing a graph-CNN model that multi-dimensionally analyzes elements and substructures that are adjacent to three-dimensional chemical structures.

## What is required for further improvement of the prediction rate?

As stated previously, the prediction accuracy for Ames mutagenicity are approximately 80 and 77% by QSAR and by SMILES-CNN, respectively. What would be required to further improve the accuracy and attain an accuracy closer to 100%? My view is that the issue is not with the prediction model, but rather with the Ames test results themselves. According to the survey of Ames test data from public domains, estimated inter-laboratory reproducibility of Ames tests is around 85% [24, 25], which is almost equivalent to the predictive power of the better QSAR or SMILES-CNN. It means that the prediction accuracy of 80% has almost reached saturation, and even if QSAR models are improved by the accumulation of more test data, further improvement of the predictive power is difficult. For the improvement of the prediction power, improvements of QSAR models as well as improvement of methodological weakness and data evaluation of Ames test on mutagenic mechanism are important. For example, Ames test results are binary, either positive or negative, meaning that a weak positive response in some cases could be judged as negative. Generally, positive results in Ames tests have a quantity equal to or greater than twice the negative control as their standard for positivity (2-fold rule) [26]. When the maximum mutation frequency is 1.9-fold with dosage dependency and repeatability, the rule renders a negative result judgment. On the other hand, there are the cases in which when there are multiple data and only one of them is positive, a conservative judgment of positive is made out without considering mutagenic mechanisms. These obscure test results were integrated into the database and used for developing QSAR models. These incorrect data hamper predictions and are a source of noise in the development of accurate QSAR models.

**Fig. 3:**
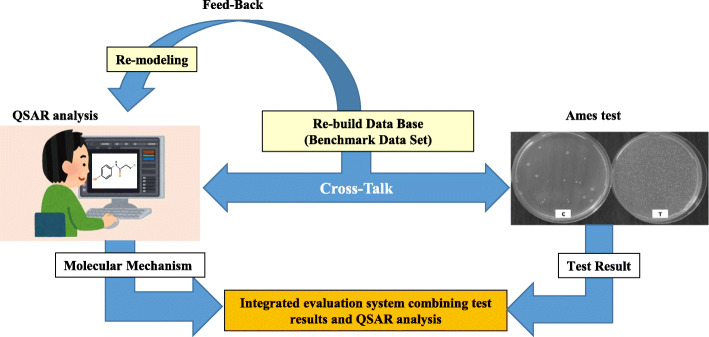
Evaluation of mutagenicity of chemicals by Integrated Approach

Whether in QSAR or AI, as long as data from biological test results are used as training data, the reliability of the data is the most important element in terms of improving the prediction power. It is thus essential to establish a large benchmark database consisting only of well-validated Ames test results to build more accurate QSAR models. Positive outcome by QSAR can sometimes suggest the alert structures and mechanisms used as the basis for judgment. If this mechanism is relevant, there is a need to question the actual test result and reassess it. Upon judging the test results, the expert judgment based on the mechanism is important, as opposed to a formulaic or conservative manner based on a guideline. Cross-talk between actual test results and QSAR results can lead to a correct assessment. From this assessment, a reliable benchmark data set is constructed, and QSAR modela can be remodeled using this data set. By adding new training data sets and repeating the process of cross-talk with test results followed by remodeling, it is expected that the predictive power of QSAR and/or AI tools can be improved continuously and that they would surpass judgment by actual test results (Fig. 3).

In reality, time is still needed for in silico predictions to surpass real tests in terms of reliability. Currently, I am recommending an integrated approach when assessing the mutagenicity of chemical substances, whereby both Ames tests and QSAR are used, and the final conclusion should be derived from the results of both types of studies (Fig. 3). The quality of test data reported through assessment reports or scientific research papers is multifarious, and such results cannot simply be trusted. The idea is to use QSAR, not as a prediction tool, but as a supporting tool for final assessment of actual test results. We are practically using this approach for assessing the mutagenicity of food flavorings as well as chemical substances eluted from plastic apparatuses/containers and packaging.

## Concluding remarks

At the beginning of this review, I mentioned that so far humans have created about 200 million chemicals. So how many chemicals can theoretically be created? It depends on the molecular weight and the type of constituent atoms, but its number from test calculations is generally estimated to be between 10^33^ and 10^60^, which is the final chemical space [27]. These numbers exceed astronomical numbers. Obviously, assessing the safety of each of these chemical substances by biological testing is absolutely impossible. Thus, it is obvious that in silico methods will definitely become important in the future. While the chemical space is estimated to be between 10^40^ and 10^60^, there is something known as the Go space, which has been calculated to be as large as 10^360^. Currently the AI program, “Alpha Go”, is competing well with human Go masters. In comparison, the chemical space is overwhelmingly small, and I am optimistic that predicting the toxicity of chemicals will be easy in the near future with AI. Now that self-driving cars are coming to life by AI, AI will be able to reliably predict the properties of immobile chemicals. The important thing is to give AI the correct toxicity information and train repeatedly. This is not an easy task, but it will be realized in the near future. During the twenty-first century, toxicology will definitely switch from biology to chemistry (I hope). And genetic toxicology will be a pioneering in in silico toxicology.

## Data Availability

Not applicable.

## Abbreviations

CAS: Chemical Abstracts Service
WSSD: World Summit on Sustainable Development
(Q)SAR: (Quantitative) Structure–Activity Relationship
ADI: Acceptable Daily Intake
PDE: Permitted Daily Exposure
ICH: International Conference on Harmonization
OECD: The Organisation for Economic Cooperation and Development
SOHN: Self-organizing hypothetical network
TIMES: Tissue Metabolite Simulator
NTP: National Toxicology Program
T.E.S.T: Toxicity Estimation Software Tool
TP: True positive
FP: false positive
FN: False negative
TN: True negative
MCC: Mathews’ correlation coefficient
MHLW: Ministry of Health Labor and Welfare
GLP: Good Laboratory Practice
NIHS: National Institute of Health Sciences
DGM: The Division of Genetics and Mutagenesis
AI: Artificial intelligence
SMILES: Simplified molecular-input line-entry system
CNN: Convolutional neural network
